# AI-Assisted Clinical Decision Support Versus Standard Teleconsultation in Adult Medicine: A Systematic Review of Diagnostic Accuracy, Referral and Testing Decisions, and Clinician Confidence

**DOI:** 10.3390/clinpract16100181

**Published:** 2026-09-30

**Authors:** Yaslam Ba Srada, Bobakr Bawazir, Saad Mohammad Aghi, Mohamed Elsayed, Hisham Yousif, Mohamed Ibrahim, Kareem Adel Eltohamy, Mark Sim

**Affiliations:** Medical Engagement Department, Health Horizon Medical Co., Jeddah 21323, Saudi Arabia; dr.bobakrbawazir@gmail.com (B.B.); saadaghi@gmail.com (S.M.A.); mohammed_am1989@hotmail.com (M.E.); hipz@ymail.com (H.Y.); bor3m28@hotmail.com (M.I.); kareem.eltohamy@gmail.com (K.A.E.); mark.rsim47@gmail.com (M.S.)

**Keywords:** artificial intelligence, clinical decision support, teleconsultation, telemedicine, diagnostic accuracy, referral, physician confidence, systematic review

## Abstract

**Background/Objectives:** Artificial intelligence (AI) is increasingly integrated into teleconsultation, but its effects on clinician performance remain uncertain. We assessed whether clinician-facing AI improves diagnostic accuracy, reduces referrals or testing, and increases clinician confidence compared with standard teleconsultation or conventional resources. **Methods:** This retrospectively registered systematic review followed the PRISMA 2020 and Synthesis Without Meta-Analysis (SWiM) guidance. PubMed/MEDLINE, publisher-indexed platforms, Google Scholar, ClinicalTrials.gov, and backward and forward citation searches were performed from inception to 1 August 2026. Eligible studies evaluated clinician-facing AI in adult primary, urgent, or general medical teleconsultation or in high-fidelity virtual consultation designs and reported diagnostic, referral/testing, or confidence outcomes. One reviewer screened records, extracted data, and assessed the risk of bias using the design-appropriate QUADAS-2, RoB 2, and ROBINS-I domains, with a second pass for consistency. Owing to clinical and methodological heterogeneity, the results were synthesized narratively. **Results:** Of 31 records identified, 25 remained after duplicate removal, 18 full texts were assessed, and six studies were included: two randomized virtual experiments, two retrospectives virtual care cohorts, one randomized crossover conversational AI study, and one provider-to-provider telemedicine case series. In a telehealth screening experiment, AI improved diagnostic prediction, but physicians given a large language model showed no significant benefit (adjusted difference, two percentage points; 95% CI, −4 to 8). Across 102,059 virtual encounters, an AI-generated differential included the clinician’s final diagnosis in 84.2%, although the reference was not independent. AI-assisted urgent care recommendations were rated optimal more often than final physician recommendations (77.1% vs. 67.1%). AI did not reduce unnecessary referrals; one experiment found more confirmatory testing requests and lower trust than with a familiar clinical score. Certainty was low or very low for the principal clinical outcomes. **Conclusions:** AI may support teleconsultation decisions, but evidence that it reduces unnecessary referrals or meaningfully increases clinician confidence is insufficient. Pragmatic trials using independent patient-level reference standards are needed. Registration: This completed review was retrospectively registered on the Open Science Framework (OSF); no protocol was published before study selection.

## 1. Introduction

The teleconsultation has moved from an emergency substitute to a permanent part of healthcare delivery. It can improve access, reduce travel and wait times, and ensure continuity of care when specialist capacity is low. The World Health Organization places digital health in the broader context of a more person-centered health system [1]. In primary care, telephone and video consultations can achieve clinical outcomes and satisfaction in comparison with face-to-face care for selected conditions, but their effectiveness may depend on the problem, mode of presentation, digital access, and the need for physical examination for specific problems and procedures [2]. In a large, multispecialty study, provisional diagnoses based on video diagnosis matched in 86.9% of cases, but the performance was not the same across different specialties and presentations [3]. While teleconsultation is often effective, diagnostic uncertainty is still a major issue. Artificial intelligence can resolve this uncertainty by building the history into an algorithm, creating differential diagnoses, identifying red flags, predicting disease probabilities, and suggesting investigations or treatments. The clinical question is not whether AI works well in isolation but whether it works in conjunction with the professionals who treat real patients. Digital symptom checkers have been shown to be inaccurate in diagnosing and triage [4]. Agreement between the AI output and the clinician’s final diagnosis is also not necessarily correct, especially if clinicians view the AI suggestion before making a decision. The social concerns involved are automation bias, alert fatigue, opaque reasoning, workflow disruption, and uncertainty over who is responsible when the clinician and algorithm disagree [5]. Early evidence of virtual care is encouraging but not consistent. In 102,059 virtual primary care encounters, clinicians selected a diagnosis in an AI-generated differential in 84.2% of cases [6]. A randomized telehealth study found that explainable AI formulated better streptococcal pharyngitis predictions than the Modified Centor Score but decreased trust and increased requests for confirmatory testing [7]. In a randomized diagnostic reasoning trial, access to a large language model did not significantly improve physician performance over standard communication resources [8,9]. However, in simulated text consultations, a conversational diagnostic AI system outperformed primary care doctors in different dimensions. In real-world AI-assisted virtual urgent care, initial AI recommendations were rated as optimal more often than the physician’s final plan [10], whereas, in a provider-to-provider telemedicine study, AI responses were more complete, but clinical accuracy was not established [11,12,13]. These results point to three outcomes that should not be conflated: diagnostic accuracy, referral or testing efficiency, and clinician confidence. In particular, accuracy may be better when referrals are higher because clinicians are more likely to distrust the new outcomes; confidence is raised but not better. Previous reviews have largely examined standalone symptom checkers or broad applications of AI and telemedicine, leaving uncertainty about clinician-facing AI used within adult teleconsultations. The present review therefore evaluated whether clinician-facing AI, compared with standard teleconsultation or conventional clinical resources, improves diagnostic accuracy, referral and testing decisions, and clinician confidence. Throughout the review, diagnostic accuracy against an independent reference standard was distinguished from concordance between AI outputs and clinician decisions.

## 2. Materials and Methods

### 2.1. Design and Reporting

We conducted a systematic review with narrative synthesis in accordance with the Preferred Reporting Items for Systematic Reviews and Meta-Analyses (PRISMA) 2020 statement [14] and the Synthesis Without Meta-Analysis (SWiM) guidelines [15]. The completed PRISMA 2020 checklist is provided in Supplementary Table S1, and the study selection process is reflected in the PRISMA 2020 flow diagram (Figure 1). The review question, eligibility criteria, outcomes, and synthesis plan were specified before study selection. The completed review was retrospectively registered on the Open Science Framework (OSF; https://doi.org/10.17605/OSF.IO/KZVXY). Registration occurred after completion of the review and should not be interpreted as prospective preregistration. No publicly accessible protocol was prepared before study selection, and there were no amendments to a prospectively registered protocol.

### 2.2. Eligibility Criteria

We used the PICOS framework (Table 1). The population comprised physicians or other licensed clinicians delivering adult primary care, urgent care, general internal medicine care, or provider-to-provider medical teleconsultation. High-fidelity virtual diagnostic encounters were eligible as supportive evidence when they reproduced a remote consultation and involved practicing clinicians. The intervention was clinician-facing AI decision support, including machine learning classifiers, explainable AI interfaces, structured AI medical interviews, differential diagnosis generators, and large language models. Eligible comparators were standard teleconsultation, standard clinical decision tools, conventional information resources, physician recommendations, or unassisted clinician performance. Primary outcomes were diagnostic accuracy against an independent reference standard, a verified final diagnosis, or a validated diagnostic reasoning rubric. Diagnostic concordance was retained but explicitly distinguished from accuracy. Secondary outcomes were referral, escalation, confirmatory testing, investigation rates, or appropriateness; clinician trust, perceived understanding, confidence, or reliance; and management quality, safety, consultation time, and workload. Randomized trials, prospective or retrospective comparative studies, diagnostic accuracy studies, and empirical case series were eligible. Reviews, editorials, purely technical model development studies, patient-only chatbots, remote monitoring without a clinical consultation, and specialty-specific imaging pathways outside general medicine were excluded. Studies were grouped for synthesis by outcome domain (diagnostic performance, referral/testing decisions, and clinician confidence) and by clinical realism (core real-world teleconsultation versus supportive virtual simulation).

### 2.3. Information Sources and Search Strategy

PubMed/MEDLINE, publisher-indexed journal platforms, Google Scholar, ClinicalTrials.gov, and the reference lists and forward citations of relevant studies and reviews were searched from database inception to 1 August 2026; each source was last searched on this date. Search concepts covered telemedicine, AI, and the prespecified outcome domains. No publication year restriction was applied. An English-language full text was required because resources for validated translation were unavailable. ClinicalTrials.gov was searched for completed or ongoing comparative studies and potential publication gaps. Appendix A reports the exact search syntax or search terms, source-specific limits, and the common last-search date. The preserved screening log recorded search yields at an aggregate level—24 records from database and publisher platform searching and seven from ClinicalTrials.gov and citation searching—rather than as separate counts for each web or publisher source. Source-specific yields for Google Scholar and individual publisher platforms were therefore not reconstructed retrospectively.

### 2.4. Selection Process

One reviewer deduplicated the records and screened all titles and abstracts; they then assessed the retrieved full-text reports against the eligibility criteria. The same reviewer repeated the eligibility assessment in a separate second pass to check consistency. No machine learning or other automation tool was used for screening. Disagreements between reviewers did not arise because screening was performed by one reviewer; this is acknowledged as a review process limitation. Each excluded full-text report was assigned one dominant exclusion reason. Studies were classified as core evidence when AI was embedded in real-world teleconsultation and as supportive evidence when a randomized or crossover virtual simulation evaluated clinician–AI diagnostic collaboration. The study selection process is shown in Figure 1.

### 2.5. Data Extraction

One reviewer extracted the data into a piloted structured form and checked the completed form against every included report in a second pass. No automation tool was used, and study investigators were not contacted. Extracted variables were the publication year, country, setting, design, sample size, clinician type, clinical presentations, teleconsultation modality, AI system and version when reported, comparator, reference standard, follow-up, funding, and conflicts of interest. Outcomes sought were diagnostic accuracy, diagnostic reasoning scores, diagnostic concordance, referral or escalation, confirmatory testing or investigation use, appropriateness, confidence, trust, reliance, management quality, safety, consultation time, and workload. We sought all eligible reported measures and time points; when several analyses addressed the same outcome, the prespecified primary or most adjusted analysis was prioritized. Numerical results were extracted as reported. Percentage-point differences were calculated only when both group proportions were available and were used descriptively rather than as a common effect measure. Missing or unclear information was recorded as not reported and was not imputed. Agreement between AI output and a clinician’s final diagnosis was labeled as concordance and was not treated as verified diagnostic accuracy.

### 2.6. Risk of Bias and Applicability

One reviewer assessed the risk of bias and applicability using design-appropriate tools and repeated the assessment in a separate consistency check. Diagnostic accuracy domains were evaluated with QUADAS-2 (patient selection, index test, reference standard, and flow and timing) [16]. Randomized studies were evaluated using RoB 2 principles [17] and non-randomized comparative evidence using ROBINS-I domains [18]. No automation tool was used. AI-specific considerations included whether the algorithm was fixed before evaluation, whether experimental advice was programmed to be correct, whether clinicians saw AI recommendations before deciding, the independence and blinding of adjudicators, the exclusion of low-confidence cases, proprietary involvement, and transferability to routine teleconsultation. Overall judgements were low risk, some concerns, serious risk, or critical risk. Because these labels spanned different instruments, domain-level judgements were used to inform rather than mechanically determine certainty ratings.

### 2.7. Synthesis and Certainty

Studies were assigned to a synthesis only when their population, AI function, comparator, and outcome matched a prespecified review domain. Results were tabulated by study and outcome, and selected absolute percentage-point differences were calculated from reported group proportions. No missing summary statistics were imputed, and no effect estimates were converted beyond these descriptive differences. Table 2, Table 3 and Table 4 summarize the study characteristics, individual results, risk of bias, and certainty; Figure 2 displays selected study-level absolute results without pooling. A meta-analysis was not conducted because the clinical conditions, AI functions, comparators, units of analysis, reference standards, and outcome definitions were too heterogeneous for a meaningful common estimate. The narrative synthesis considered the direction of effect, magnitude, precision, clinical realism, and risk of bias. A formal subgroup analysis, meta regression, and sensitivity analysis were not performed because only six heterogeneous studies were eligible. Reporting bias was considered qualitatively by comparing outcomes described in the methods with the reported results and by searching ClinicalTrials.gov, but funnel plots and statistical tests were not used because fewer than ten studies contributed to every outcome and effects were not pooled. Certainty was rated by outcome using the GRADE principles for risk of bias, inconsistency, indirectness, imprecision, and publication bias [19]. Randomized evidence started at high certainty and observational evidence at low certainty, with downgrading where appropriate. Methodological expectations for diagnostic studies were informed by PRISMA-DTA [20].

## 3. Results

### 3.1. Study Selection and Characteristics

The searches identified 31 records: 24 from databases and publisher platforms and seven from ClinicalTrials.gov and citation searching. Six duplicates were removed, leaving 25 records for title and abstract screening. Seven records were excluded at screening. All 18 reports sought for retrieval were obtained and assessed in full-text form; 12 were excluded because they concerned the wrong specialty (*n* = 4), did not evaluate teleconsultation (*n* = 3), were secondary or non-empirical reports (*n* = 3), or lacked an eligible outcome (*n* = 2). Six studies (six reports) were included in the narrative synthesis (Figure 1): four core teleconsultation studies and two supportive high-fidelity virtual diagnostic studies. Selected reports that appeared eligible but were excluded are identified with reasons in Appendix B. The evidence comprised 102,900 clinical encounters or scenarios and 191 clinicians, with one retrospective cohort contributing most encounters. Publication dates ranged from 2023 to 2026. AI functions included automated history-taking, differential diagnosis, explainable image-supported prediction, large language model diagnostic support, and diagnosis and management recommendations. Table 1 presents the review framework, and Table 2 summarizes the study characteristics.

### 3.2. Diagnostic Accuracy and Decision Quality

Evidence for a diagnostic benefit was mixed. The AI-supported interfaces improved disease state prediction in the randomized telehealth strep throat study of 121 providers [7]. The AI advice in the experimental cases was aimed at being correct, so we cannot deduce performance in cases where the diagnosis was wrong or uncertain. This study demonstrates the potential value of AI as a method of presentation, but no real-world model accuracy is established. In the 102,059 virtual primary care encounters, clinicians selected a diagnosis within the AI differential in 84.2% of the total encounters and selected the top-ranked AI diagnosis in 60.9% [6]. For 35 diagnoses (at least 95% of encounters), the diagnosis was agreed on by reviewers, and, for 57 diagnoses, at least 90% of reviewers agreed on the AI differential. In the cases where reviewers reached an agreement, the diagnosis of common agreement was always in the AI differential. However, the clinician’s choice was not an independent reference standard, and the clinician had access to the AI output; hence, the main estimate is concordance rather than unbiased diagnostic accuracy. As a result, the randomized trial of 50 healthcare providers—predominantly internal medicine physicians—reported no significant improvement in diagnostic reasoning when physicians used GPT-4 instead of conventional resources. The median diagnostic reasoning score was 76% for a large language model and 74% for conventional resources, with an adjusted difference of two percentage points (95% CI −4 to 8; *p* = 0.60). The model alone was 16 percentage points above the physician group using conventional resources, showing that good standalone model performance does not translate into better human–AI team performance. In the conversational AI crossover study, AMIE was compared to 20 primary care physicians in 159 text-based scenarios [9]. Specialist assessors rated AI as more accurate and better in 30 of the 32 clinical dimensions, while patients were more favorable towards it in 25 of 26 of the clinical dimensions. The new text-only interface and the patient actors in the simulation made it challenging to perform video or telephone calls. In 461 real-world AI-assisted virtual urgent care visits, the initial AI recommendations were in agreement with the final physician recommendations in 56.8% of cases, while only 67.1% of doctors were. Independent reviewers rated AI recommendations as the best in 77.1% of cases, while the physician was rated the best in 11.3%, so the AI was more accurate in 20.8% of cases and the physician was better in 11.3% of cases. AI was more likely to detect red flags, suggest indicated tests or referrals, and adhere to guidelines; physicians were more prepared to integrate information and avoid unnecessary emergency referrals. The non-blinded source presentation, the exclusion of low-confidence AI cases, and the unknown physician exposure to the AI results increased the bias. The provider-to-provider case series compared volunteer physician and AI responses to 50 asynchronous consultations from African clinicians [11]. AI responses more often contained a diagnosis list (82% vs. 24%), next diagnostic steps (100% vs. 84%), immediate treatment recommendations (100% vs. 58%), red flags (52% vs. 8%), and follow-up concerns (74% vs. 18%). These outcomes do not reflect diagnostic success or a patient benefit but the quality of the overall system. The outcomes are shown in Table 3.

### 3.3. Referral, Escalation, and Confirmatory Testing

No study reported a reduction in unnecessary referrals if one set an independent appropriateness standard. In the strep throat experiment, AI improved prediction, but more confirmatory testing was requested for negative cases. Compared to a mean testing rate of 0.30 according to the Centor Score, the rates were 0.45 for performance characteristic explanations and 0.51 for feature contribution explanations [7]. The urgent care study suggested that AI could prevent omissions of indicated investigations and referrals, but it also noted situations in which doctors more appropriately avoided emergency department referrals [10]. As the study compared recommendations (not referrals) and not referrals made, the net referral efficiency was not measured. The provider-to-provider study more frequently presented diagnostic steps and red flags but did not evaluate whether the recommendations were necessary, followed, or beneficial [11].

### 3.4. Physician Confidence, Trust, and Usability

Confidence-related evidence was less robust. Only the telehealth strep throat experiment directly measured perceived trust. The mean trust was 4.95 for the familiar Centor Score, 4.65 for the AI performance characteristic interface, and 4.62 for the feature contribution interface [7]. The adjusted decreases were 0.41 and 0.24 for trust, and example explanations did not affect trust. Higher AI agreement also coincided with lower subjective trust and more testing. Other studies reported clinician–AI agreement, response quality, or workflow observations but did not provide a controlled confidence measure.

### 3.5. Risk of Bias and Certainty

Figure 3 shows the sample sizes, clinical realism, and risk of bias. The randomized physician trial had the most credible comparative design but was still indirect regarding routine teleconsultation. The strep throat experiment was not without risk, because AI recommendations were always correct in AI cases. The conversational AI trial was randomized but blinded and simulated. In both real-world virtual care cohorts, the risk of bias was high due to non-independent comparators, selection, decision-making, and commercial involvement. The small telemedicine case series had serious-to-critical limitations for causal inference. The level of certainty of evidence was low for improvements in diagnostic performance and very low for referral/testing outcomes and clinician confidence. The main reasons were as follows: indirectness of findings, non-independent reference standards, a lack of outcome reporting, and imprecision. Table 4 provides the study-level risk-of-bias judgments and outcome-level certainty ratings. Figure 2 presents selected non-pooled absolute differences; we emphasize that the outcomes are not commensurable.

## 4. Discussion

This systematic review found a credible but low-certainty signal that AI can augment certain aspects of diagnostic work in teleconsultation. This study did not show that AI-based teleconsultation was truly better than standard care. Clinical and methodological heterogeneity was substantial across AI functions, clinical settings, comparators, reference standards, and outcome definitions. Consequently, the studies should not be interpreted as estimating one common effect of AI-assisted teleconsultation, and their findings have limited comparability and generalizability. The evidence is more contextually oriented: AI was best when generating a broad differential, looking for red flags, and reducing omissions; it was less helpful when clinicians had to deal with evolving narratives, reconcile contradictory information, decide on escalation and how much to escalate, and how to translate the model output into a patient-centered plan. This interpretation is consistent with the methodological caution of the PRISMA, SWiM, and diagnostic test reporting frameworks, which discourage the use of heterogeneous proxies if they are related to one clinical effect [14,15,20]. The difference between accuracy and concordance is crucial. In the largest study, an AI differential featured the clinician’s final diagnosis in 84.2% of visits [6]. This can be useful in practice because such a broad differential is a cognitive forcing technique. However, the clinician had seen the AI output, and the final diagnosis was not regularly checked by an external reference standard. Hence, the high concordance could be due to model quality, clinician adoption of AI recommendations, or both. QUADAS-2 and ROBINS-I indicate that non-independent reference standards and post-index incorporation can significantly increase performance [16,18]. In future research, we should consider the AI recommendation, the clinician’s unaided decision, the post-AI decision, and the independently adjudicated reference diagnosis separately. The randomized evidence also indicates that a good model is not necessarily a better human–AI team. Access to GPT-4 did not substantially improve doctors’ diagnostic reasoning scores in the Goh trial [8]. This collaboration gap may be due to poor prompting, limited time, failure to recognize relevant advice, automation bias, or reluctance to follow unfamiliar advice. Data from human–computer studies show that clinical practitioners can improve with appropriate AI but are still susceptible to confidently presented incorrect advice, and answers to questions do not guarantee good team performance [21,22,23]. The assessment of the interface design, uncertainty communication, and clinician training should be part of the intervention and not optional. Referral and testing results were lower than diagnostic outcomes. AI did not reduce unnecessary referrals; no study proved this. The best comparative result was in the opposite direction: clinicians desired more confirmatory testing when negative AI predictions were presented, likely because they trusted the AI less than the familiar Centor Score [7]. This does not mean that more testing is always undesirable. A cautious clinician may expect to receive confirmation when a negative result is very expensive or the model is not transparent to the outside world. However, this finding offers a good example of how diagnostic accuracy and resource use can differ. Referral reduction should be based on appropriate criteria and not simply on a decrease in referrals. The virtual urgent care cohort provides a more complementary message: AI recommendations were rated as optimal more often and were particularly capable of identifying red flags and missing investigations [10]. Physicians, however, were better able to avoid unnecessary emergency referrals and incorporate new information in some cases. These findings generate the hypothesis that AI could serve as an auditable safety net that checks for omissions and guideline deviations while clinicians retain authority over contextual interpretation; this role was not directly tested in the included studies. This is consistent with broader evidence that AI trials are heterogeneous and that clinical benefit is linked to workflow integration as opposed to benchmark performance alone [13,24]. Confidence should not be taken as a given outcome. Appropriate confidence is calibrated confidence: it is high when the combined evidence is reliable and lower when data are incomplete, the model is not in its field of validation, or the clinician and AI disagree. The reduction in trust despite better predictions might be attributed to reasonable skepticism, poor interface design, unfamiliarity, or a lack of calibration [7]. Future trials should test confidence before and after AI access, agreement with correct and incorrect advice, override behavior, responses to model uncertainty, and whether confidence predicts actual accuracy. CONSORT-AI and SPIRIT-AI need to be designed with a clear understanding of the intended use, user interaction, error handling, and human oversight in mind [25,26]. Achieving generalizability and equity is also important. Proprietary systems were tested in selected digital populations, and the African provider-to-provider study evaluated completeness over outcomes [11]. Models based on data from high-income, English-speaking individuals may be inaccurate when the symptom descriptions, disease prevalence, available studies, and clinical roles differ. A pragmatic cluster-randomized primary care trial in Kenya conducted in 2026 found LLM assistance to be safe but did not significantly reduce 14-day treatment failure despite improvements in process measures [27]. This highlights the need to understand better documentation as the difference between better patient experiences of treatment and better outcomes in the clinical setting, as well as the need to predict subgroups based on age, sex, language, multimorbidity, clinical complexity, and digital access. The low and very-low certainty ratings do not mean that AI is ineffective. They indicate that the current estimates are unreliable and often answer a narrower question than those posed by health systems. A registered randomized trial in El Salvador is directly comparing AI-enabled and standard telemedicine with diagnostic accuracy as an outcome and may fill part of this gap [28]. Future pragmatic trials should report actual referrals, emergency transfers, investigations, treatment changes, adverse events, repeat contacts, time to resolution, patient experiences, costs, and clinician workloads. AI should be employed as auditable decision support with explicit escalation rules, version control, subgroup monitoring, and continuous algorithm vigilance in place to avoid clinical judgment being compromised.

### 4.1. Limitations

This review has limitations. The evidence base was small, heterogeneous, and dominated by proprietary systems, simulated encounters, and retrospective comparisons. The English-language restriction may have excluded relevant evidence. The review was not prospectively registered, and no public protocol was prepared before study selection; however, the completed review was retrospectively registered on OSF (https://doi.org/10.17605/OSF.IO/KZVXY) to improve transparency. Screening, extraction, and risk-of-bias assessment were completed by one reviewer, although each was checked in a separate second pass; selection and extraction errors therefore remain possible. The search included broad web and publisher platform sources, which are less reproducible than bibliographic databases. The original search documentation did not preserve a defensible source-by-source retrieval count for Google Scholar or individual publisher platforms; only the verified aggregate source counts used in the PRISMA flow were available. A meta-analysis was inappropriate because the outcome definitions and comparators differed substantially. Referral appropriateness and confidence were rarely measured, and several studies used concordance or structural completeness rather than independently validated diagnostic accuracy or patient outcomes. With only six heterogeneous studies, reporting bias, subgroup effects, and robustness could not be assessed statistically.

### 4.2. Strengths

The review separated diagnostic accuracy from concordance, referral efficiency, and clinician confidence rather than combining them into a single claim of effectiveness. It integrated real-world virtual care with clearly labeled supportive simulations, applied design-appropriate risk-of-bias domains, followed the PRISMA and SWiM principles, and graded certainty. Study-level effects were presented without inappropriate statistical pooling, preserving clinically important differences in AI function, workflow, comparator, and reference standards.

## 5. Conclusions

AI-assisted decision support may improve differential diagnosis coverage, red flag recognition, and the completeness of recommendations during teleconsultation. However, it has not been shown to consistently improve clinician diagnostic performance, reduce unnecessary referrals, or increase physician confidence. The certainty of evidence for the principal outcomes was low or very low due to indirectness, heterogeneity, non-independent reference standards, and imprecision. The findings suggest that an auditable safety-net role is a potential implication for future evaluation, rather than an effect established by the included studies. Future multicenter pragmatic trials should compare AI-assisted and standard teleconsultation using blinded independent adjudication and should measure actual referrals and tests, downstream diagnoses, adverse events, confidence calibration, workloads, and costs. Algorithms should be externally validated, version-locked during evaluation, monitored for subgroup performance and drift, and designed to communicate uncertainty and trigger human review when confidence is low.

## Figures and Tables

**Figure 1:**
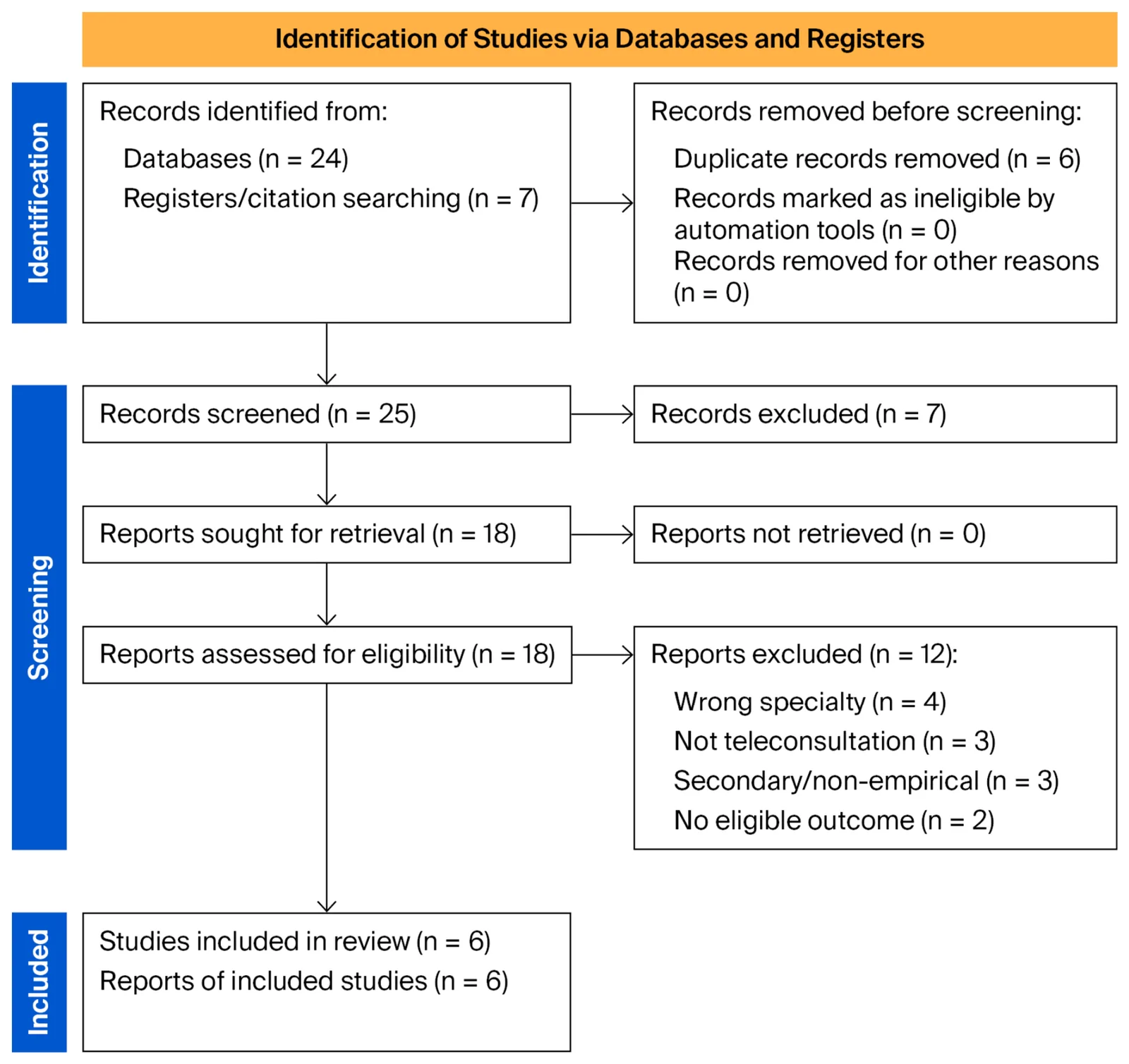
PRISMA 2020 flow diagram of study identification, screening, eligibility assessment, and inclusion. Note: Counts were verified against the final screening log and database exports.

**Figure 2:**
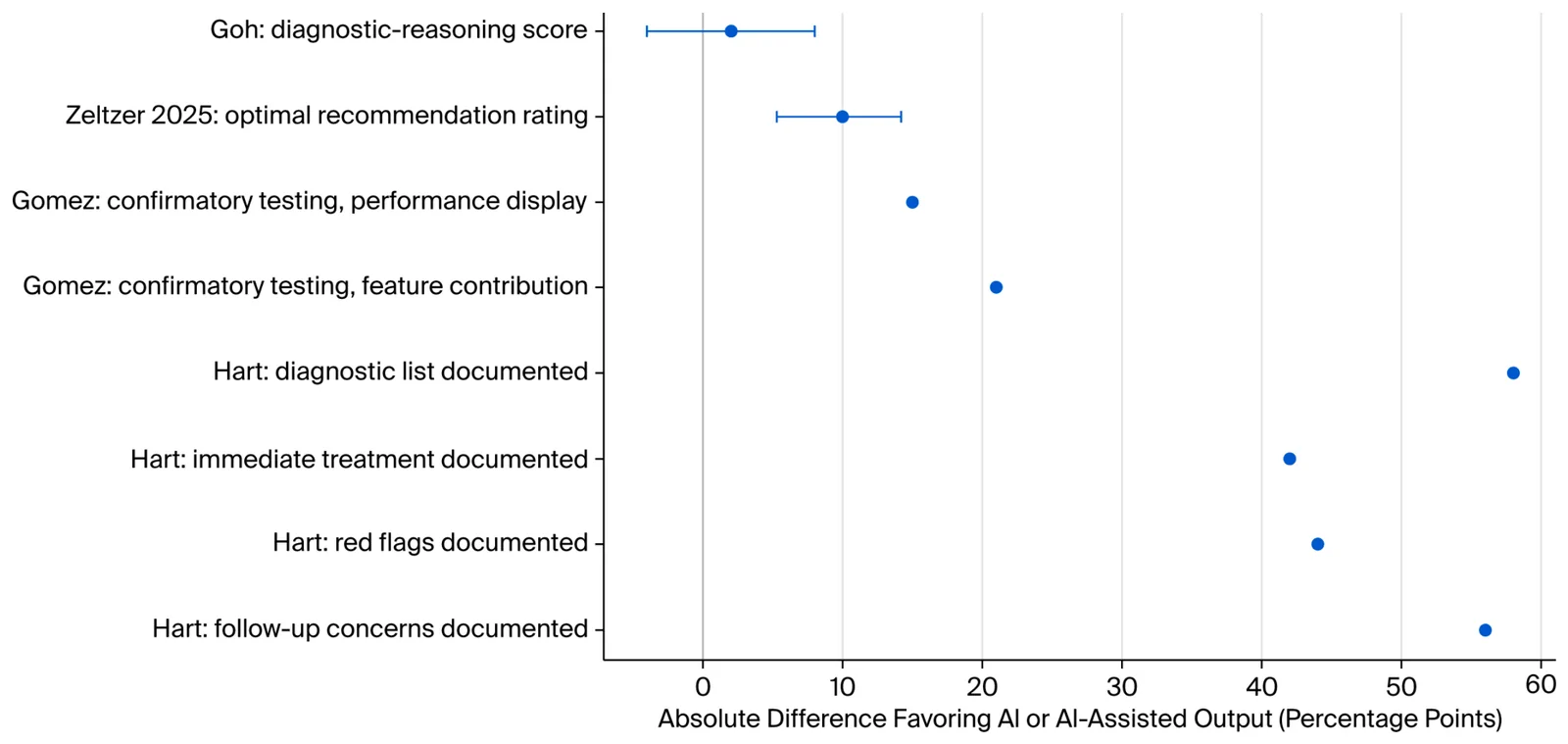
Selected study-level effects presented descriptively without pooling. Points show reported absolute study results; horizontal intervals are displayed only when the source study reported an uncertainty interval. Outcomes use different scales and are not directly comparable.

**Figure 3:**
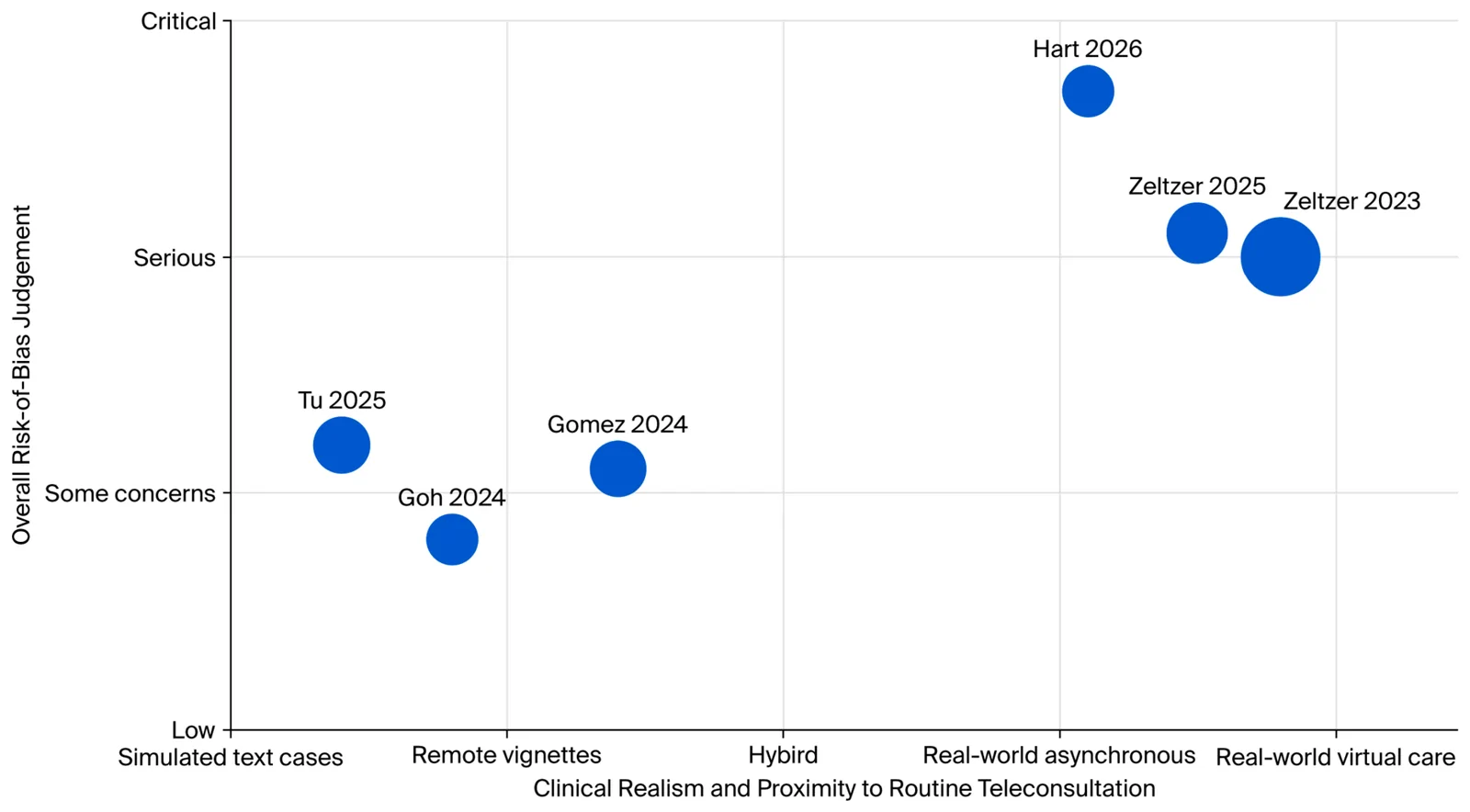
Evidence map of clinical realism, study scale, and risk of bias. Bubble size represents the study sample size; horizontal position represents proximity to routine teleconsultation; vertical position represents overall risk of bias [6,7,8,9,10,11].

**Table 1:** Review question, eligibility criteria, and outcome framework.

Domain	Eligibility Specification
Population	Licensed clinicians delivering adult primary care, urgent care, general internal medicine, or provider-to-provider medical teleconsultation; high-fidelity virtual consultations included as supportive evidence.
Intervention	Clinician-facing AI decision support: machine learning classifier, explainable AI, structured AI interview, differential generator, or large language model.
Comparator	Standard teleconsultation, conventional clinical tool or information resource, physician recommendation, or unassisted clinician performance.
Primary outcome	Diagnostic accuracy against an independent reference standard or validated rubric; concordance retained but labeled separately.
Secondary outcomes	Referral, escalation, testing, appropriateness, clinician confidence or trust, management quality, safety, consultation time, and workload.
Study designs	Randomized trials, diagnostic accuracy studies, comparative prospective or retrospective studies, and empirical case series.
Exclusions	Patient-only chatbots; remote monitoring without consultation; technical model development; non-empirical reports; specialty imaging outside general medicine.

**Table 2:** Characteristics of included studies.

Study	Design and Setting	Participants/Encounters	AI Intervention	Comparator	Main Outcomes	Key Limitations
Zeltzer et al., 2023 [6]	Retrospective virtual primary care cohort, USA	102,059 encounters	Structured AI interview and differential diagnosis	Treating clinician final diagnosis	Differential contained clinician diagnosis in 84.2%; top-ranked match 60.9%	Final diagnosis not independent; clinicians viewed AI; commercial platform
Gomez et al., 2024 [7]	Randomized telehealth screening experiment, USA	121 providers	Explainable AI interfaces for strep throat prediction	Modified Centor Score	Improved prediction; increased confirmatory testing; lower trust	AI advice constructed as correct; vignette-based; narrow condition
Goh et al., 2024 [8]	Multicenter randomized vignette trial, USA	50 physicians	GPT-4 plus conventional resources	Conventional resources	Adjusted diagnostic reasoning difference +2 points (95% CI −4 to 8)	Clinical vignettes rather than live teleconsultations; possible learning effects
Tu et al., 2025 [9]	Randomized double-blind crossover remote OSCE	20 PCPs; 159 scenarios	AMIE conversational diagnostic AI	Primary care physicians	AI superior for diagnostic accuracy and most dialog domains	Simulated patients; unfamiliar text chat; developer-led study
Zeltzer et al., 2025 [10]	Retrospective AI-assisted virtual urgent care cohort, USA	461 visits	Initial AI diagnosis and management recommendations	Final physician recommendations	Optimal: 77.1% AI vs. 67.1% physician; concordance 56.8%	Adjudicators not blinded; low-confidence cases excluded; exposure uncertain
Hart, 2026 [11]	Retrospective provider-to-provider telemedicine case series, Africa–USA	50 consultations	OpenEvidence-generated response	Volunteer physician response	AI responses more structurally complete across all measured elements	No accuracy standard, outcomes, or adjustment; small sample; single AI prompt

**Table 3:** Main findings by outcome domain.

Outcome Domain	Study	AI-Assisted Result	Comparator Result	Effect/Interpretation
Diagnostic concordance	Zeltzer 2023 [6]	AI differential contained final clinician diagnosis: 84.2%	No unaided comparator	High concordance; not independently verified accuracy
Diagnostic reasoning	Goh 2024 [8]	Median 76%	Median 74%	Adjusted difference +2 percentage points (95% CI −4 to 8); not significant
Conversational diagnostic performance	Tu 2025 [9]	Superior on 30/32 specialist-rated axes	Primary care physicians	Strong simulated performance; substantial indirectness
Recommendation quality	Zeltzer 2025 [10]	77.1% rated optimal	67.1% rated optimal	Absolute difference +10.0 points; adjudication not blinded
Confirmatory testing	Gomez 2024 [7]	0.45 to 0.51 testing rate in negative cases	0.30 with Centor Score	AI increased testing by 15 to 21 points
Trust	Gomez 2024 [7]	Mean 4.62 to 4.65	Mean 4.95	Lower trust despite improved prediction
Response completeness	Hart 2026 [11]	Diagnosis list 82%; red flags 52%; follow-up 74%	24%; 8%; 18%	More complete documentation; clinical correctness unknown
Unnecessary referral	All studies	Not established	Not established	No direct evidence of reduction using an appropriateness standard

**Table 4:** Risk-of-bias judgements and certainty of evidence.

Study/Outcome	Selection	AI/Index Test	Reference Standard/Comparator	Flow, Blinding, Confounding	Overall Judgement	GRADE Certainty
Zeltzer 2023 [6]	Some concerns	Some concerns	Serious	Serious	Serious risk	Low for concordance; very low for accuracy
Gomez 2024 [7]	Low	Serious: advice always correct	Some concerns	Some concerns	Some concerns to serious	Low
Goh 2024 [8]	Low	Low	Low	Some concerns: vignette design	Some concerns	Moderate for simulated reasoning; low for teleconsultation
Tu 2025 [9]	Low	Some concerns: developer system	Low	Serious indirectness	Some concerns	Low
Zeltzer 2025 [10]	Serious	Some concerns	Serious: unblinded adjudication	Serious: exposure/ selection/confounding	Serious risk	Very low
Hart 2026 [11]	Serious	Some concerns	Critical: no truth standard	Serious	Critical for comparative effectiveness	Very low
Diagnostic performance (overall)	-	-	-	Inconsistent and indirect	-	Low
Referral/testing (overall)	-	-	-	Sparse, indirect, no appropriateness standard	-	Very low
Clinician confidence (overall)	-	-	-	Single controlled study	-	Very low

## Data Availability

No new data were created or analyzed in this study.

## Supplementary Materials

The following supporting information can be downloaded at: https://www.mdpi.com/article/10.3390/clinpract16100181/s1, Supplementary Table S1: PRISMA 2020 checklist.

## Abbreviations

AI, artificial intelligence; AMIE, Articulate Medical Intelligence Explorer; GRADE, Grading of Recommendations Assessment, Development, and Evaluation; LLM, large language model; OSCE, objective structured clinical examination; PRISMA, Preferred Reporting Items for Systematic Reviews and Meta-Analyses; QUADAS-2, Quality Assessment of Diagnostic Accuracy Studies 2; RoB 2, Risk of Bias 2; ROBINS-I, Risk of Bias in Non-Randomized Studies of Interventions; SWiM, Synthesis Without Meta-Analysis.

## Appendix A

### Appendix A.1. Search Strategies, Search Dates, Limits, and Yield Documentation

All sources were last searched on 1 August 2026. No publication year limit was used; an English-language full text was required. The source-specific search syntax and limits are provided below.

PubMed/MEDLINE: ((telemedicine[Title/Abstract] OR telehealth[Title/Abstract] OR teleconsult*[Title/Abstract] OR “virtual care”[Title/Abstract] OR “remote consultation”[Title/Abstract]) AND (“artificial intelligence”[Title/Abstract] OR “machine learning”[Title/Abstract] OR “large language model*”[Title/Abstract] OR LLM[Title/Abstract] OR “clinical decision support”[Title/Abstract] OR “explainable AI”[Title/Abstract]) AND (diagnos*[Title/Abstract] OR “diagnostic accuracy”[Title/Abstract] OR “diagnostic reasoning”[Title/Abstract] OR referral*[Title/Abstract] OR triage[Title/Abstract] OR testing[Title/Abstract] OR investigation*[Title/Abstract] OR confidence[Title/Abstract] OR trust[Title/Abstract])) AND humans[Filter].

Google Scholar: allintitle: (telemedicine OR telehealth OR teleconsultation OR “virtual care”) (“artificial intelligence” OR “large language model” OR “clinical decision support”) (diagnosis OR referral OR testing OR confidence OR trust). The first 200 results sorted by relevance were reviewed.

ClinicalTrials.gov: Condition/disease: adult primary care OR urgent care OR internal medicine; Other terms: (telemedicine OR telehealth OR teleconsultation) AND (artificial intelligence OR machine learning OR large language model OR clinical decision support); Study type: interventional and observational; Status: all.

Publisher-indexed journal platforms: targeted full-text searches used the PubMed concept blocks adapted to each available platform interface: (telemedicine OR telehealth OR teleconsultation OR “virtual care”) AND (“artificial intelligence” OR “large language model” OR “clinical decision support”) AND (diagnosis OR referral OR testing OR confidence OR trust). The final search log did not preserve a reliable platform-by-platform yield, so no retrospective platform-specific counts were reconstructed.

Supplementary searching: reference lists of included studies and relevant reviews were screened, and forward citation searches were performed in Google Scholar. Duplicates were removed manually. No automated screening tool was used.

### Appendix A.2. Search Yield Documentation

The final screening log verified 31 records before deduplication: 24 from database and publisher platform searching and seven from ClinicalTrials.gov and citation searching. Six duplicates were removed, leaving 25 records for title and abstract screening. The original documentation did not retain a defensible source-by-source split among PubMed/MEDLINE, Google Scholar, ClinicalTrials.gov, citation searching, and individual publisher platforms. To avoid retrospective fabrication, unrecorded source-level yields were not reconstructed; only the verified aggregate counts used in the PRISMA flow are reported.

## Appendix B. Selected Full-Text Reports Excluded After Eligibility Assessment

The full-text exclusion totals were as follows: wrong specialty (*n* = 4), did not evaluate teleconsultation (*n* = 3), secondary or non-empirical report (*n* = 3), and no eligible outcome (*n* = 2). This resulted in a total of 12 exclusions. The reports listed below are the specifically cited excluded records that could appear eligible or were retained as contextual or emerging evidence.

Tschandl et al. [21]—excluded because it evaluated a specialty dermatologic imaging pathway rather than general medical teleconsultation.

Gaube et al. [22]—excluded because it evaluated susceptibility to clinical decision aids outside a teleconsultation pathway.

Bansal et al. [23]—excluded because it was a human–AI collaboration experiment outside adult medical teleconsultation.

Agweyu et al. [27]—excluded from the eligible synthesis because it evaluated in-person primary care decision support rather than teleconsultation; it was retained as contextual evidence in the Discussion.

ClinicalTrials.gov NCT07406919 [28]—excluded from the synthesis because it was an ongoing trial without published outcome data; it was retained to describe emerging evidence.

## References

[1] World Health Organization (2021). Global Strategy on Digital Health 2020–2025.

[2] Carrillo de Albornoz S., Sia K.L., Harris A. (2022). The effectiveness of teleconsultations in primary care: Systematic review. Fam. Pract..

[3] Demaerschalk B.M., Pines A., Butterfield R., Haglin J.M., Haddad T.C., Yiannias J., Colby C.E., TerKonda S.P., Ommen S.R., Bushman M.S. (2022). Assessment of clinician diagnostic concordance with video telemedicine in an integrated multispecialty practice. JAMA Netw. Open.

[4] Wallace W., Chan C., Chidambaram S., Hanna L., Iqbal F.M., Acharya A., Normahani P., Ashrafian H., Markar S.R., Sounderajah V. (2022). The diagnostic and triage accuracy of digital and online symptom checker tools: A systematic review. npj Digit Med..

[5] Kueper J.K. (2023). Complexities and questions toward artificial intelligence for diagnostic support in virtual primary care. Mayo Clin. Proc. Digit Health.

[6] Zeltzer D., Herzog L., Pickman Y., Steuerman Y., Ber R.I., Kugler Z., Shaul R., Ebbert J.O. (2023). Diagnostic accuracy of artificial intelligence in virtual primary care. Mayo Clin. Proc. Digit Health.

[7] Gomez C., Smith B.L., Zayas A., Unberath M., Canares T. (2024). Explainable AI decision support improves accuracy during telehealth strep throat screening. Commun. Med..

[8] Goh E., Gallo R., Hom J., Strong E., Weng Y., Kerman H., Cool J.A., Kanjee Z., Parsons A.S., Ahuja N. (2024). Large language model influence on diagnostic reasoning: A randomized clinical trial. JAMA Netw. Open.

[9] Tu T., Schaekermann M., Palepu A., Saab K., Freyberg J., Tanno R., Wang A., Li B., Amin M., Cheng Y. (2025). Towards conversational diagnostic artificial intelligence. Nature.

[10] Zeltzer D., Kugler Z., Hayat L., Brufman T., Ber R.I., Leibovich K., Beer T., Frank I., Shaul R., Goldzweig C. (2025). Comparison of initial artificial intelligence and final physician recommendations in AI-assisted virtual urgent care visits. Ann. Intern Med..

[11] Hart P. (2026). International telemedicine program: Physician versus AI responses. Telehealth Med. Today.

[12] Perez K., Wisniewski D., Ari A., Lee K., Lieneck C., Ramamonjiarivelo Z. (2025). Investigation into application of AI and telemedicine in rural and underserved communities: A systematic review. Healthcare.

[13] Kelly C.J., Karthikesalingam A., Suleyman M., Corrado G., King D. (2019). Key challenges for delivering clinical impact with artificial intelligence. BMC Med..

[14] Page M.J., McKenzie J.E., Bossuyt P.M., Boutron I., Hoffmann T.C., Mulrow C.D., Shamseer L., Tetzlaff J.M., Akl E.A., Brennan S.E. (2021). The PRISMA 2020 statement: An updated guideline for reporting systematic reviews. BMJ.

[15] Campbell M., McKenzie J.E., Sowden A., Katikireddi S.V., Brennan S.E., Ellis S., Hartmann-Boyce J., Ryan R., Shepperd S., Thomas J. (2020). Synthesis without meta-analysis (SWiM) in systematic reviews: Reporting guideline. BMJ.

[16] Whiting P.F., Rutjes A.W.S., Westwood M.E., Mallett S., Deeks J.J., Reitsma J.B., Leeflang M.M.G., Sterne J.A.C., Bossuyt P.M.M., QUADAS-2 Group (2011). QUADAS-2: A revised tool for the quality assessment of diagnostic accuracy studies. Ann. Intern Med..

[17] Sterne J.A.C., Savovic J., Page M.J., Elbers R.G., Blencowe N.S., Boutron I., Cates C.J., Cheng H.Y., Corbett M.S., Eldridge S.M. (2019). RoB 2: A revised tool for assessing risk of bias in randomised trials. BMJ.

[18] Sterne J.A.C., Hernan M.A., Reeves B.C., Savović J., Berkman N.D., Viswanathan M., Henry D., Altman D.G., Ansari M.T., Boutron I. (2016). ROBINS-I: A tool for assessing risk of bias in non-randomised studies of interventions. BMJ.

[19] Guyatt G.H., Oxman A.D., Vist G.E., Kunz R., Falck-Ytter Y., Alonso-Coello P., Schünemann H.J. (2008). GRADE: An emerging consensus on rating quality of evidence and strength of recommendations. BMJ.

[20] McInnes M.D.F., Moher D., Thombs B.D., McGrath T.A., Bossuyt P.M., Clifford T., Cohen J.F., Deeks J.J., Gatsonis C., Hooft L. (2018). Preferred Reporting Items for a Systematic Review and Meta-analysis of Diagnostic Test Accuracy Studies: PRISMA-DTA. JAMA.

[21] Tschandl P., Rinner C., Apalla Z., Argenziano G., Codella N., Halpern A., Janda M., Lallas A., Longo C., Malvehy J. (2020). Human-computer collaboration for skin cancer recognition. Nat. Med..

[22] Gaube S., Suresh H., Raue M., Merritt A., Berkowitz S.J., Lermer E., Coughlin J.F., Guttag J.V., Colak E., Ghassemi M. (2021). Do as AI say: Susceptibility in deployment of clinical decision-aids. npj Digit Med..

[23] Bansal G., Nushi B., Kamar E., Lasecki W.S., Weld D.S., Horvitz E. (2021). Does the whole exceed its parts? The effect of AI explanations on complementary team performance. CHI Conference on Human Factors in Computing Systems.

[24] Lam T.Y.T., Cheung M.F.K., Munro Y.L., Lim K.M., Shung D., Sung J.J.Y. (2022). Randomized controlled trials of artificial intelligence in clinical practice: Systematic review. Lancet Digit Health.

[25] Liu X., Rivera S.C., Moher D., Calvert M.J., Denniston A.K. (2020). Reporting guidelines for clinical trial reports for interventions involving artificial intelligence: The CONSORT-AI extension. BMJ.

[26] Rivera S.C., Liu X., Chan A.W., Denniston A.K., Calvert M.J. (2020). Guidelines for clinical trial protocols for interventions involving artificial intelligence: The SPIRIT-AI extension. BMJ.

[27] Agweyu A., Mwaniki P., Menon V., Korom R., Isaaka L., Wanyama C., Gill J., Kiptinness S., Adan N., Emmanuel-Fabula M. (2026). Generative AI-enabled clinical decision support system in primary care: A pragmatic, cluster-randomized trial. Nat. Med..

[28] ClinicalTrials.gov (2026). AI Telemedicine Support for Primary Care Physicians in El Salvador.

